# Lutembacher syndrome in a middle‐aged woman in Ghana with an extremely large atrial septal defect

**DOI:** 10.1002/ccr3.6939

**Published:** 2023-02-10

**Authors:** Yaw Adu‐Boakye, Gordon Manu Amponsah, Henry Kofi Andoh, Kwadwo Faka Gyan, Yaw Amo Wiafe

**Affiliations:** ^1^ Department of Medicine Kwame Nkrumah University of Science and Technology Kumasi Ghana; ^2^ Department of Medicine Komfo Anokye Teaching Hospital Kumasi Ghana; ^3^ Department of Physiology Kwame Nkrumah University of Science and Technology Kumasi Ghana; ^4^ Department of Medical Diagnostics Kwame Nkrumah University of Science and Technology Kumasi Ghana

**Keywords:** atrial septal defect, echocardiography, Lutembacher syndrome, mitral stenosis, pulmonary hypertension

## Abstract

Lutembacher syndrome (LS) is a rare heart disorder characterized by a congenital or acquired combination of the atrial septal defect (ASD) and mitral stenosis. In LS, patients may be asymptomatic for years, but early detection and treatment result in a better prognosis. In contrast, the prognosis is usually poor, with conservative treatment if the diagnosis is late and the patient develops heart failure and pulmonary hypertension. Although rheumatic heart disease (RHD) and congenital heart disease are prevalent in Ghana, cases of LS are not reported. Here, we report the case of a 45‐year‐old woman with rheumatic mitral valve stenosis and an exceptionally large ASD with bidirectional flow who was diagnosed with LS and treated conservatively for heart failure at a cardiology clinic in Ghana.

## 1 INTRODUCTION

Lutembacher syndrome (LS) is a rare clinical disorder and is characterized by the presence of a combination of congenital or acquired ostium secundum atrial septal defect (ASD) and congenital or acquired mitral stenosis (MS).^1^ Johann Friedrich Meckel, an anatomist, first identified this syndrome in 1750, and Lutembacher fully characterized it in 1916.^2^, ^3^ Although congenital ASD, in combination with acquired rheumatic ASD, was initially described for this syndrome, later definitions also included congenital MS and acquired ASD.

LS can occur at any age, but it is most common in young women.^4^ ASD is estimated to have a prevalence of 56 per 100,000 live births, accounting for 7%–10% of cases of congenital heart disease (CHD) in adults; however, congenital MS is uncommon (0.6% of total CHD cases).^5^, ^6^, ^7^ MS in LS is predominantly acquired, with rheumatic heart disease (RHD) accounting for approximately half of the cases.^1^ The actual LS prevalence is unknown; however, its occurrence may be likely in regions where RHD is common, assuming that the congenital ASD incidence remains constant. Although RHD is common in sub‐Saharan Africa (15–20 cases per 1000 individuals), only a few LS cases have been reported in the region.^8^ In this case report, we present the case of a middle‐aged Ghanaian woman who visited a cardiology clinic with heart failure and was eventually diagnosed with LS, with an exceptionally large ASD on echocardiography.

## 2 CASE PRESENTATION

A 45‐year‐old woman presented to the cardiology clinic with a 2‐year history of fatigue and progressively worsening breathlessness. She was classified as New York Heart Association‐Class III with accompanying bipedal swelling, palpitations, pink frothy sputum production, and a 1‐year history of abdominal distention. She was admitted with orthopnea and paroxysmal nocturnal dyspnea, but not chest pain. She had previously been admitted to a district hospital for spells of severe dyspnea. She had no history of diabetes, hypertension, myocardial infarction, stroke, thyroid disease, chronic kidney disease, chronic liver disease, or asthma. She had never undergone any cardiac surgery or percutaneous intervention. She had no family history of heart disease or sudden cardiac death. She is a small‐scale farmer, married with two children, and does not drink alcohol, smoke cigarettes, or consume any recreational drugs.

Physical examination revealed stage 3 finger clubbing and pitting bipedal edema up to the knee. On presentation, her blood pressure was 121/77 mm Hg, pulse rate was 103 beats per minute, and rhythm and volume were irregular. The JVP was elevated to 8 cm. Her precordium was hyperactive, with the apex beat displaced to the 7th left intercostal space in the anterior axillary line, accompanied by a thrill and left parasternal heave. She had a split‐second heart sound and a loud P2. She had both a low‐pitched rumbling mid‐diastolic murmur and a high‐pitched holosystolic murmur at the apex radiating to the axilla as well as an ejection systolic murmur at the left upper sternal boundary. A chest examination revealed fine bibasal‐end inspiratory crackles. Her abdomen was distended with demonstrable ascites and a soft, tender liver measuring 8 cm below the right costal margin.

**FIGURE 1:**
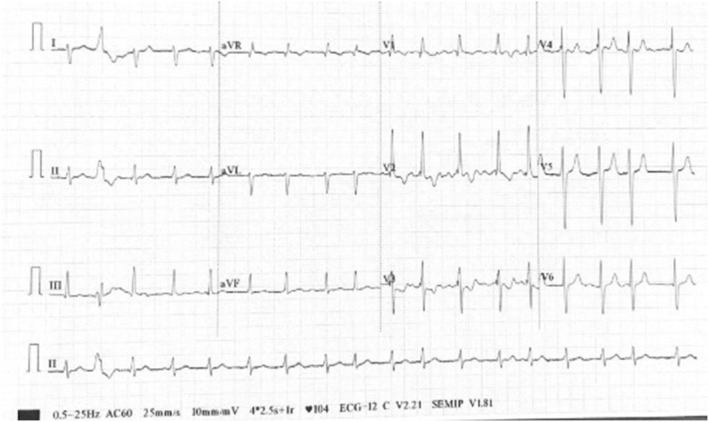
Resting electrocardiogram showing atrial fibrillation with premature ventricular contraction, right axis deviation, incomplete right bundle branch block, and right ventricular hypertrophy.

A 12‐lead electrocardiogram showed atrial fibrillation with fast ventricular response (104 beats per minute), premature ventricular complexes, right axis deviation, incomplete right bundle branch block, and right ventricular hypertrophy (Figure 1). Chest radiography showed cardiomegaly (cardiothoracic ratio, 0.69) and evidence of pulmonary congestion.

**FIGURE 2:**
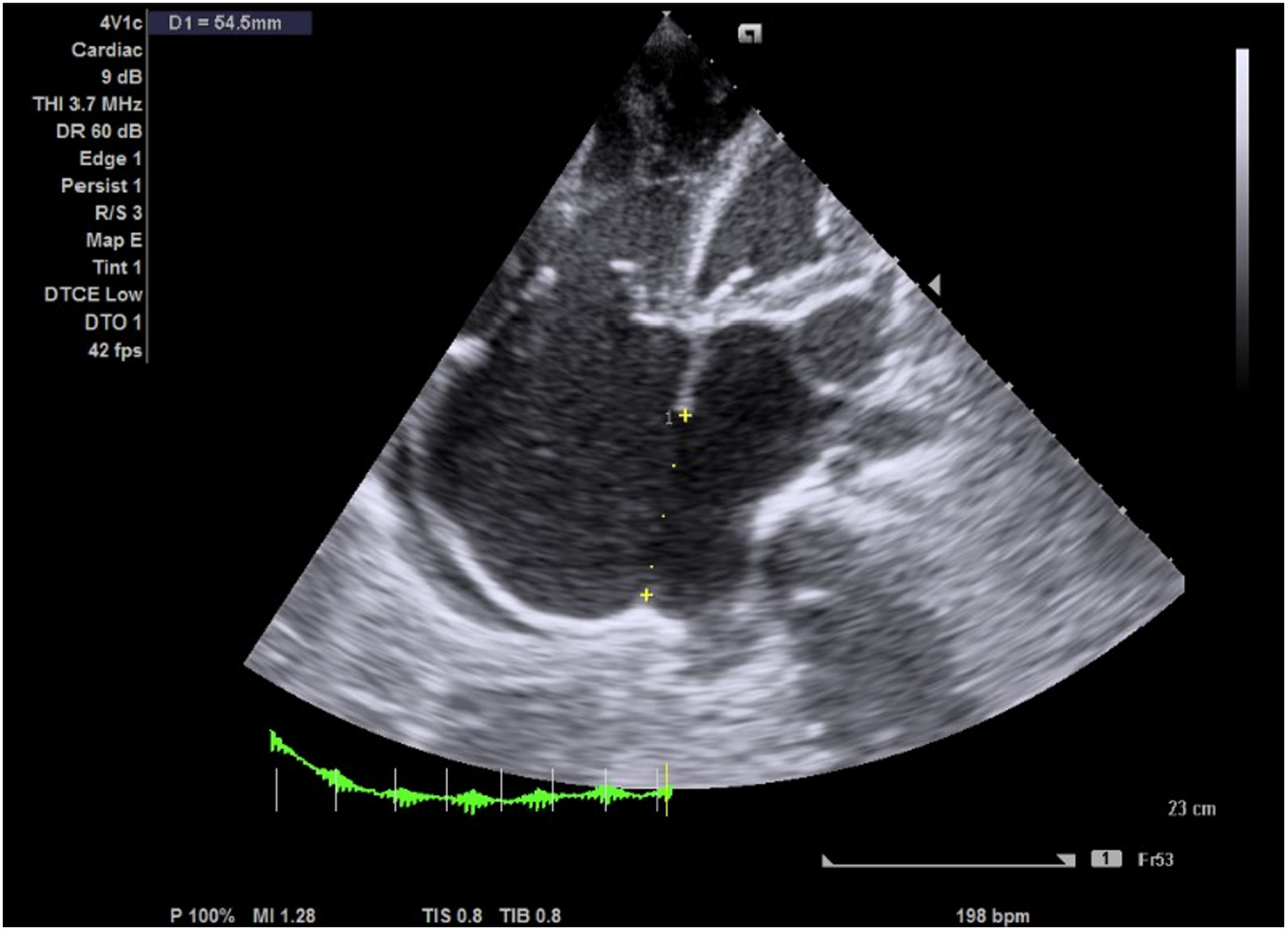
Epical 4 chamber echocardiogram showing a large atrial septal defect of size 5.45 cm and dilated right and left atria.

**FIGURE 3:**
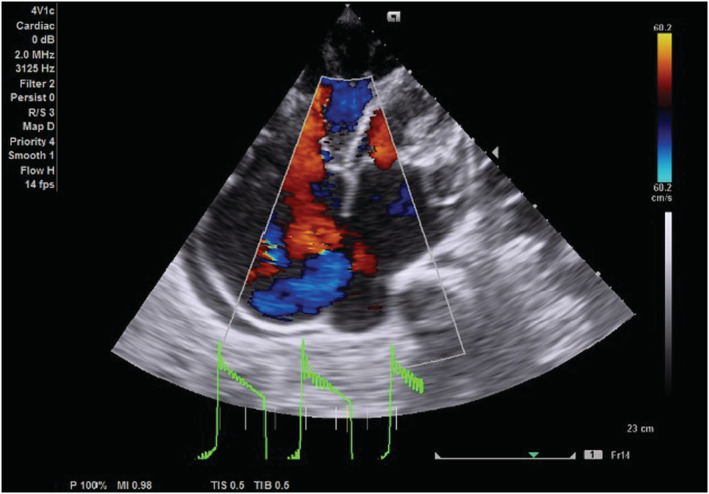
Epical 4 chamber color Doppler echocardiogram showing a large atrial septal defect with bidirectional flow.

**FIGURE 4:**
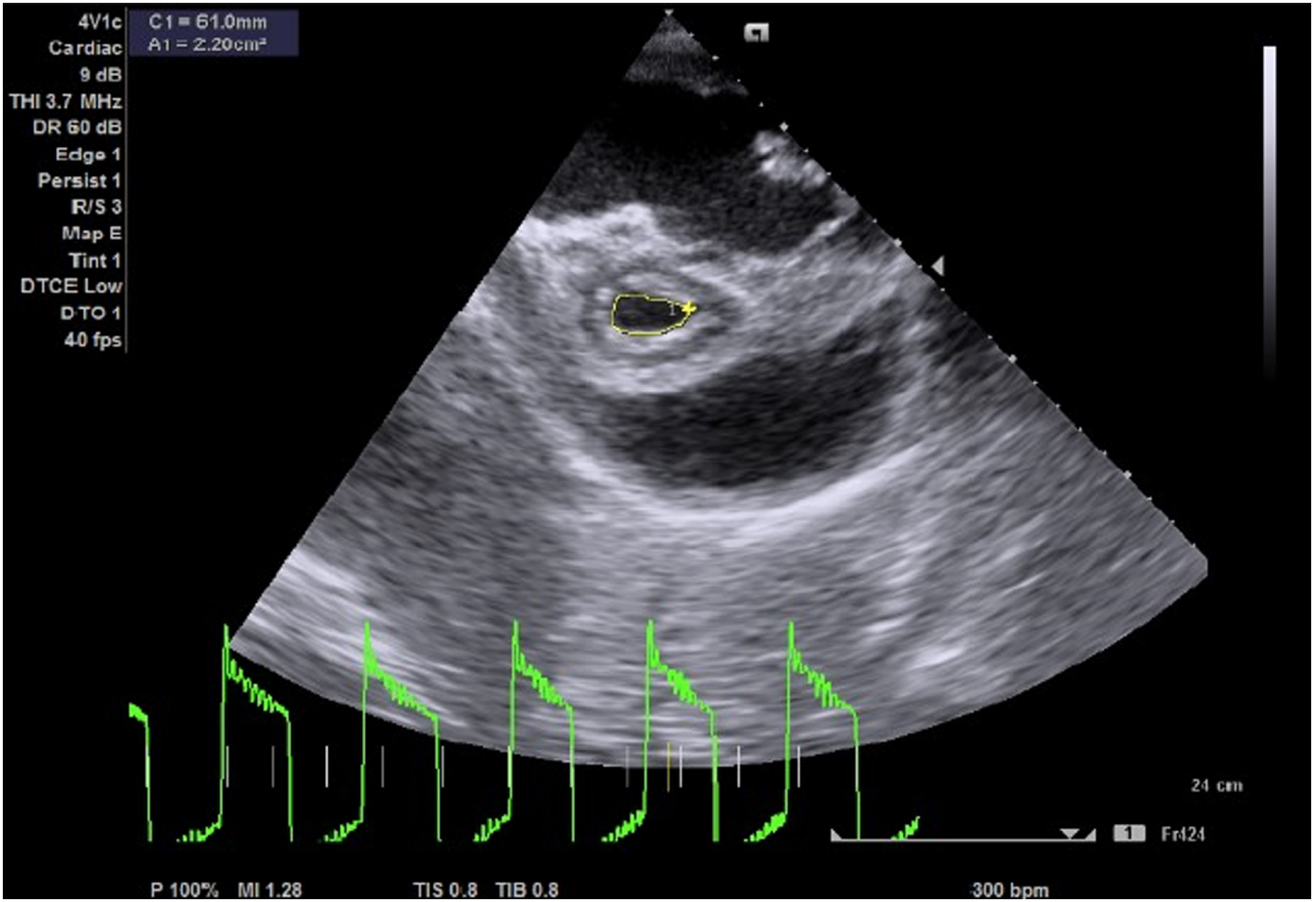
Parasternal short axis view (PSAX) echocardiogram showing mitral valve planimetry with mitral valve area of 2.2 cm^2^.

**FIGURE 5:**
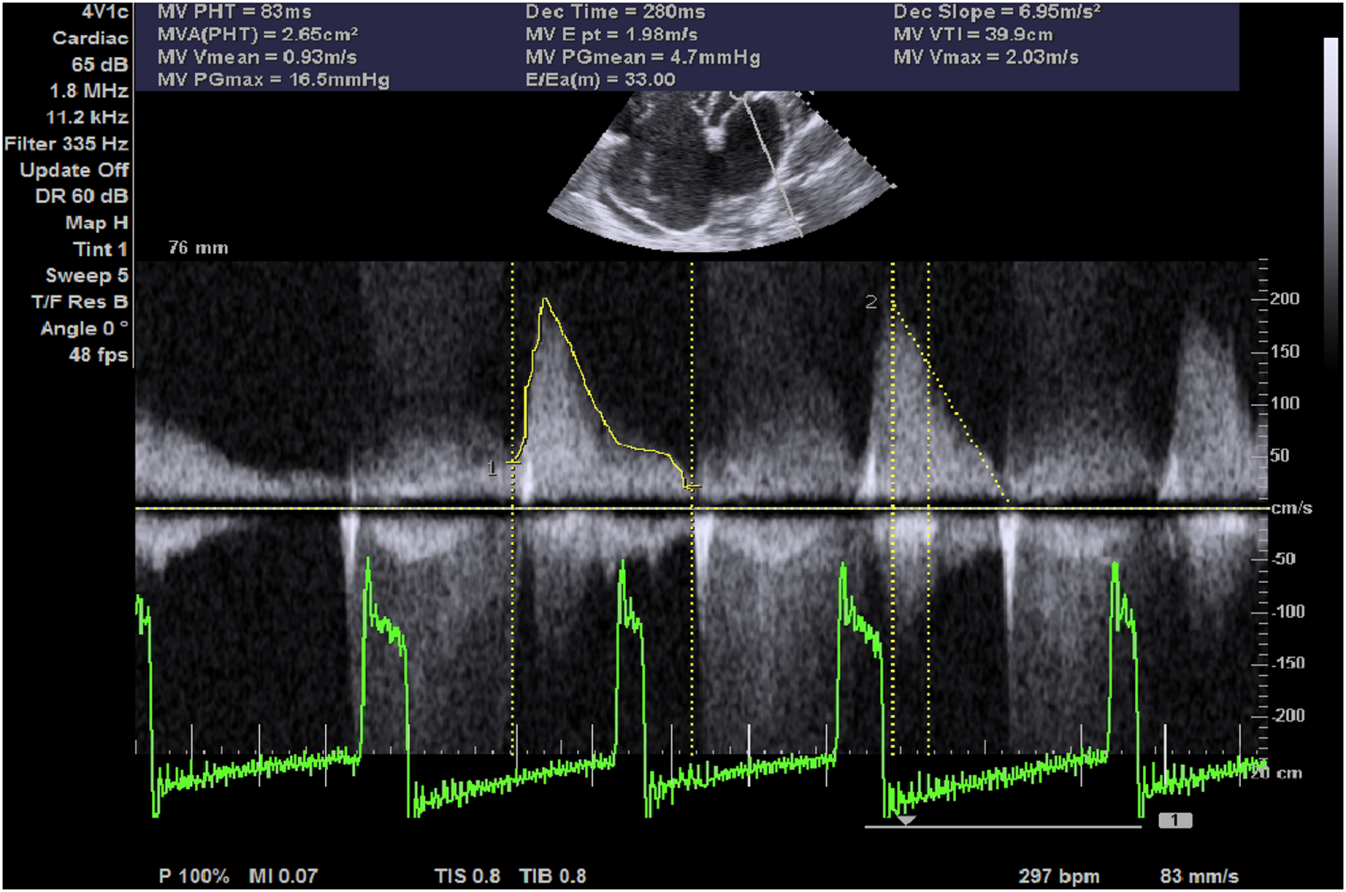
Epical 4 chamber echocardiogram showing continuous wave Doppler across the mitral valve with pressure halftime measurement and estimated mitral valve area. MAV PHT, mitral valve area derived from pressure half time; MV VTI, mitral valve velocity time integral; MVPGmax, mitral valve peak gradient; MVPHT, mitral valve pressure halftime.

**FIGURE 6:**
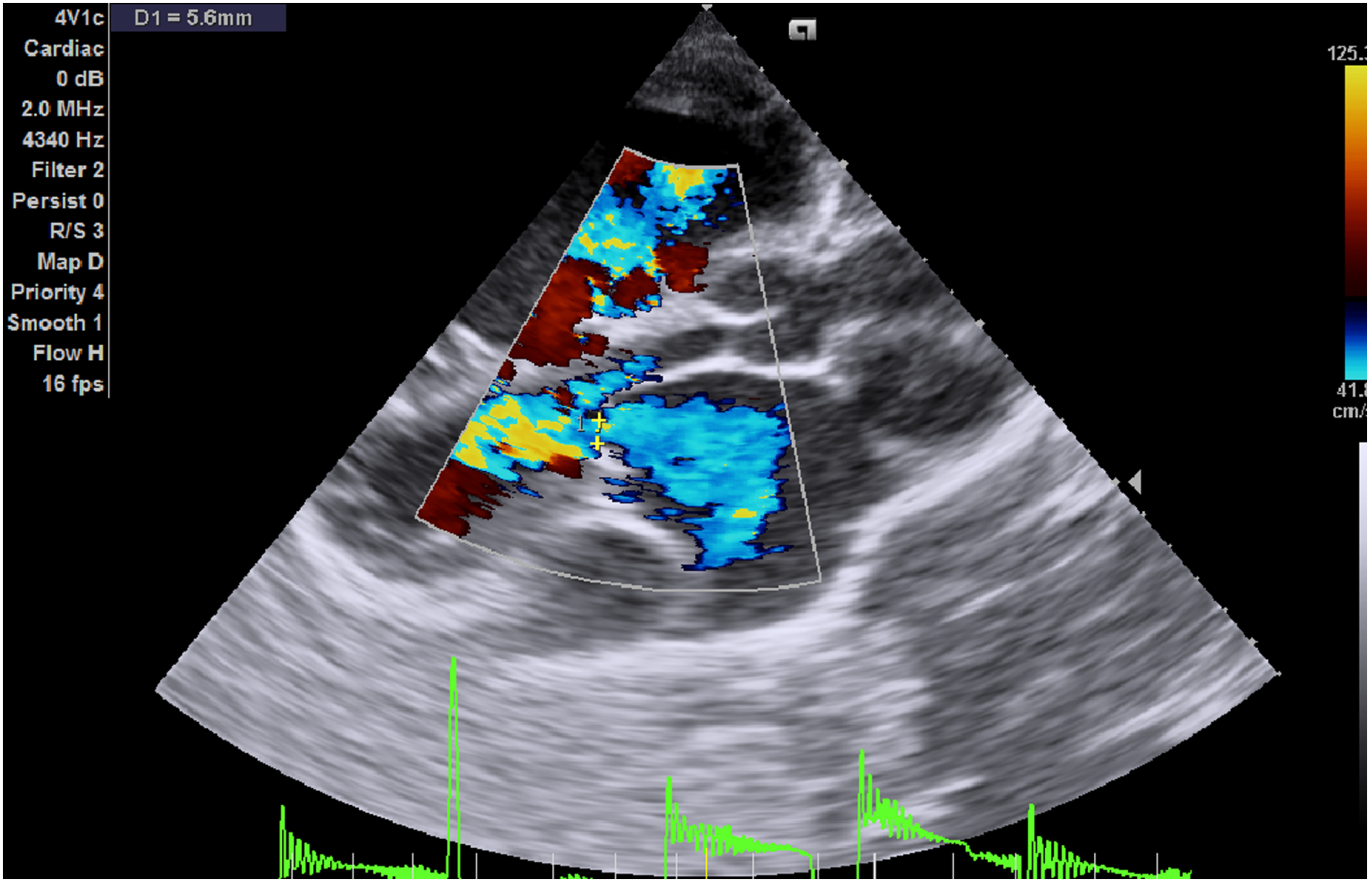
Epical 4 chamber color echocardiograph showing mitral regurgitation with vena contracta of 0.56 cm.

**FIGURE 7:**
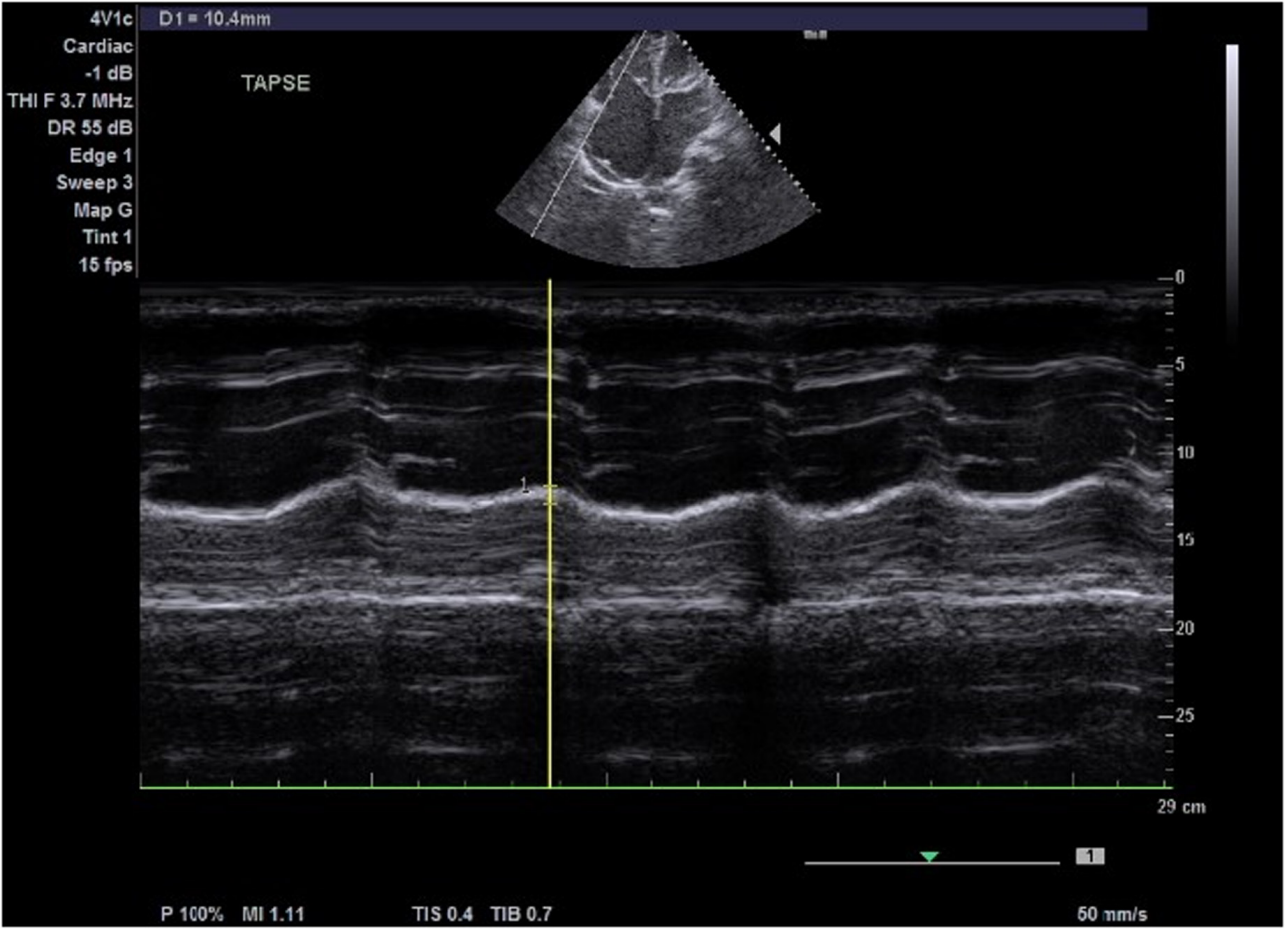
M‐mode echocardiogram showing tricuspid annular plane systolic excursion of 10.4 mm.

**FIGURE 8:**
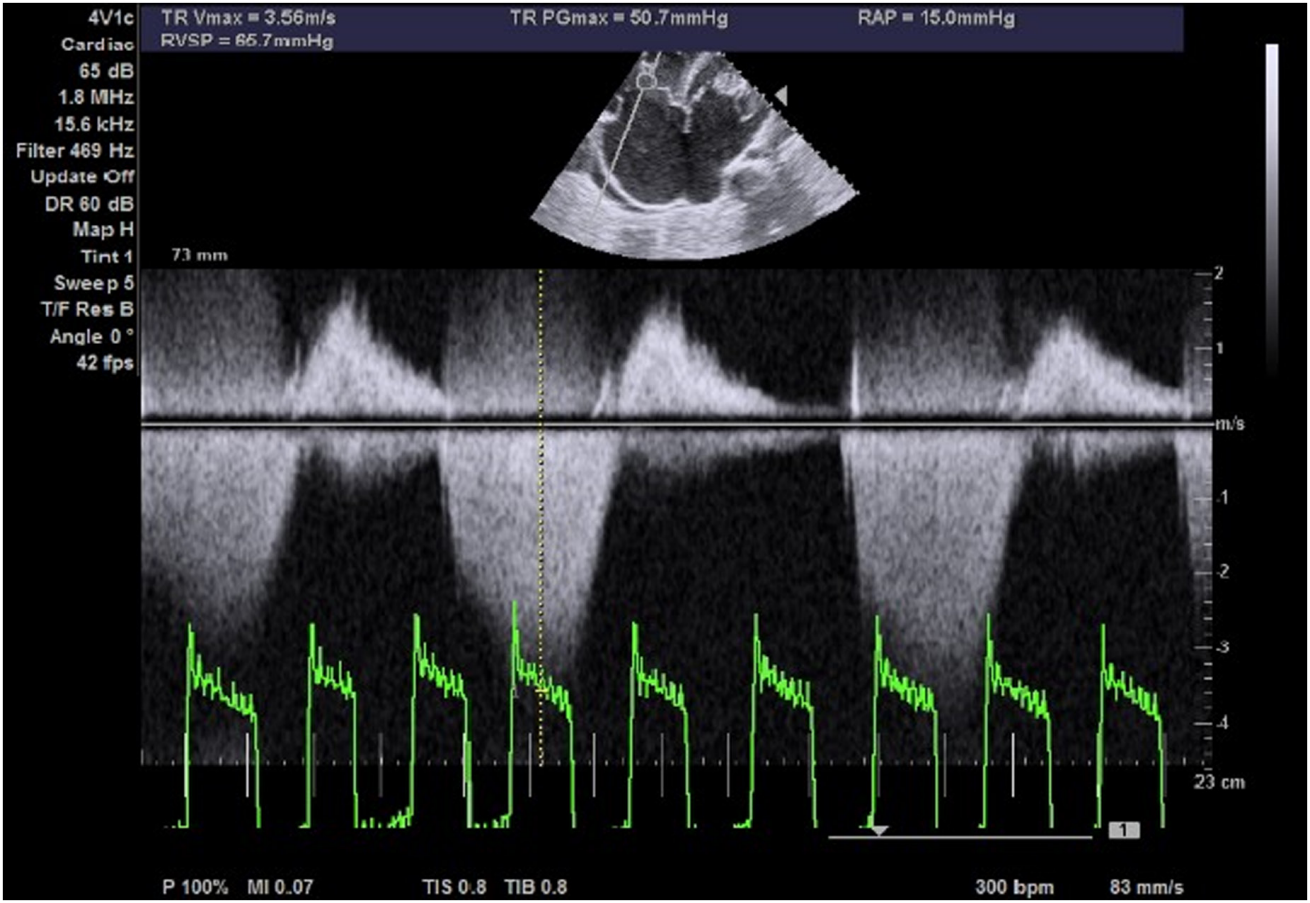
Epical 4 chamber echocardiogram showing continuous wave Doppler across the tricuspid valve revealing tricuspid regurgitation with right ventricular systolic pressure of 65.7 mm Hg.

**FIGURE 9:**
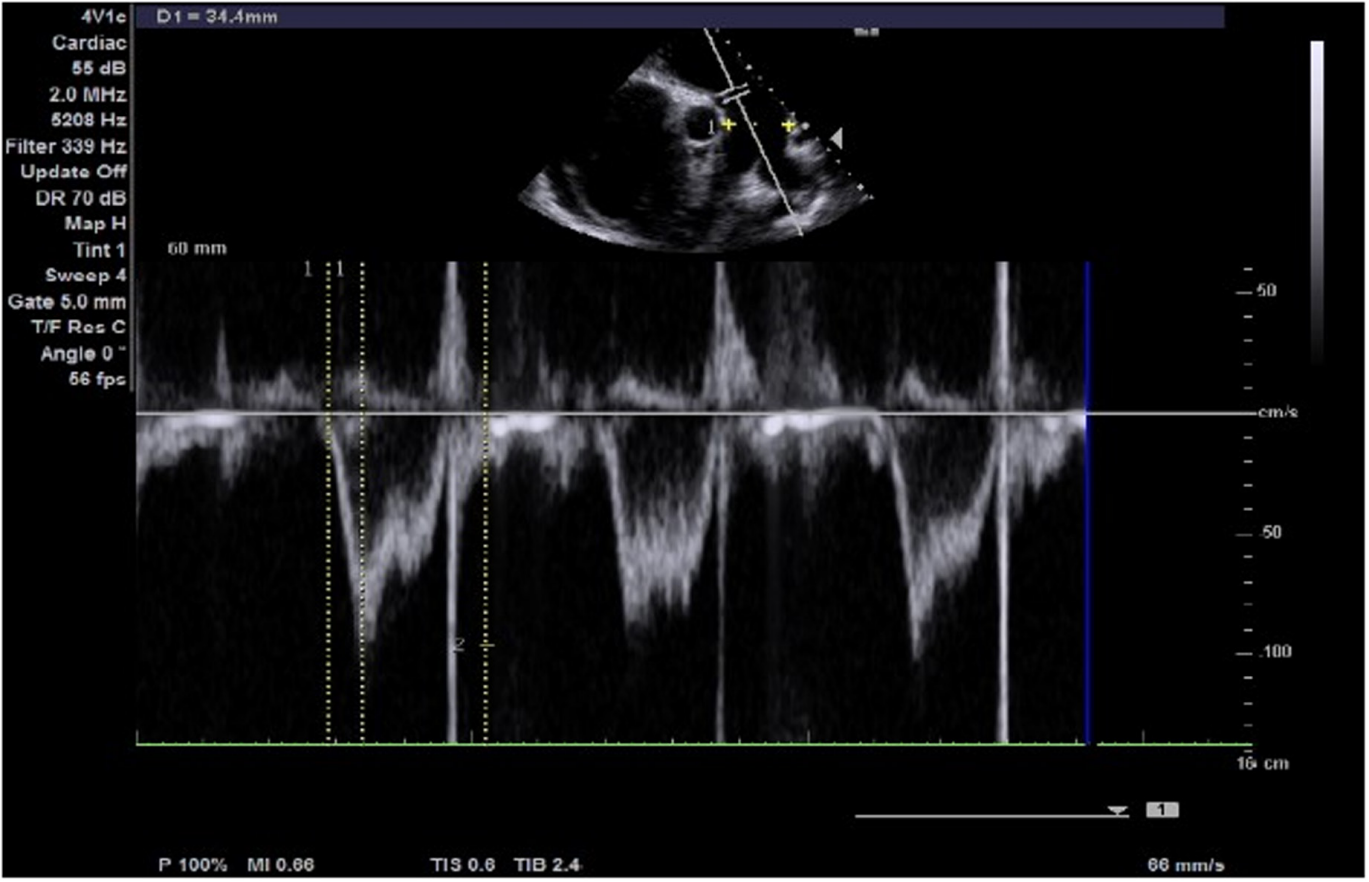
Pulse wave Doppler image showing dilated pulmonary artery of diameter (D1) 34.4 mm, normal pulmonary valve peak gradient of 1 m/s, and shortened pulmonary artery acceleration time of 96 ms.

On transthoracic echocardiography, the left and right atria were significantly dilated, and a large ASD measuring 5.45 cm (Figure 2) with the bidirectional flow was observed (Figure 3). The left ventricle had normal dimensions and systolic function. The mitral valve leaflets were thickened and demonstrated restricted mobility with a mitral valve area of 2.2 cm^2^ on planimetry (Figure 4) and 2.65 cm^2^ from the pressure half‐time measurement (Figure 5). Color Doppler examination showed retrograde flow, extending into the left atrium beyond one‐third, with a vena contractor of 0.56 cm (Figure 6), which was consistent with moderate mitral regurgitation (MR). In comparison to the left ventricle, the right ventricle was relatively dilated with reduced systolic function (tricuspid annular plane systolic excursion = 10.4 mm, Figure 7). The tricuspid valve had normal morphology; however, there was significant tricuspid regurgitation (maximum tricuspid regurgitation velocity at 3.56 m/s) and a dilated inferior vena cava (3.3 cm) that collapsed <50% during inspiration, providing an estimated right ventricular systolic pressure of 65.7 mm Hg (Figure 8). The pulmonary valve had normal morphology and peak velocity but shortened pulmonary artery acceleration time (96 ms). The main pulmonary artery was dilated (diameter of 34.4 cm) together with its branches (Figure 9). These findings are consistent with severe pulmonary hypertension. The aortic valve had normal morphology and function. Mild pericardial effusion was also observed. Abdominal ultrasound showed hepatomegaly, hepatic venous congestion, and moderate ascites. Her full blood count, liver function tests, blood urea nitrogen, creatinine, thyroid function test, lipid profile, hepatitis B surface antigen, hepatitis C antibody, and human immunodeficiency virus test results were unremarkable.

The patient was diagnosed with LS (ASD with rheumatic MS) with moderate mitral regurgitation, atrial fibrillation, and Eisenmenger syndrome. Surgical intervention was not considered due to the poor outcomes related to Eisenmenger syndrome, and the patient was conservatively treated with furosemide, losartan, spironolactone, bisoprolol, prophylaxis for bacterial endocarditis, and warfarin (anticoagulant). A significant improvement has been observed in the patient's clinical symptoms after 3 months.

## 3 DISCUSSION

Patients with LS are often asymptomatic for many years as observed in this case, and the patients may live for up to 80 years before experiencing symptoms of cardiac decompensation.^9^ The clinical presentation and natural history of this disease are influenced by the severity of MS, ASD size, pulmonary vascular resistance, and right ventricular compliance.^7^, ^10^ ASDs larger than 1.5 cm are considered significant^11^; however, the ASD measured in this case was extremely large (5.45 cm); to the best of our knowledge, the largest described for LS.

As expected, our patient presented with fatigue and exercise intolerance due to the less systemic blood flow caused by both the MS and the left‐to‐right shunting of blood across the ASD in diastole, limiting blood flow to the left ventricle. The shunting of blood through the ASD prevents pulmonary congestion in the early stages of the disease, but predisposes to the development of right atrial dilation, causing a risk of atrial arrhythmias. Palpitations caused by atrial fibrillation are common symptoms.

In individuals with non‐restrictive ASD, symptoms of pulmonary congestion usually appear later, but in patients with restrictive ASD and moderate‐to‐severe MS, symptoms of pulmonary congestion may appear much earlier.^10^ The prognosis for patients with LS is generally favorable, particularly in those who are detected early and do not have pulmonary hypertension. Although surgery is an option, current percutaneous transcatheter treatments for MS and ASD closure in the LS are preferred.^7^, ^11^, ^12^ The prognosis is poor if a patient is diagnosed late (as was the case in this report) and pulmonary hypertension has occurred. In such conditions, only conservative treatment is recommended to alleviate symptoms, control heart failure, and reduce the risk of subacute bacterial endocarditis.^13^, ^14^

Economic constraints and a less developed health system in sub‐Saharan Africa may cause delays in LS diagnosis and treatment, as demonstrated in our case. Echocardiography remains the modality of choice for the diagnosis and evaluation of LS.^12^ In this case report, both 2D and color Doppler were highly useful for detecting ASD with the bidirectional flow. A high concordance was also observed between 2D planimetry and pressure half‐time measurements in detecting MS. Vena contracta measurements helped determine the severity of mitral regurgitation.

## 4 CONCLUSION

The prognosis of LS is worse when a large ASD is complicated by pulmonary hypertension (Eisenmenger syndrome) and heart failure. Transthoracic echocardiography is useful not only for early detection but also for severity assessment in determining the best management strategy. Efforts to improve access to echocardiography in regions endemic to RHD could minimize the late detection of other associated cardiac pathologies.

## AUTHOR CONTRIBUTIONS

**Yaw Adu‐Boakye:** Conceptualization; supervision; writing – review and editing. **Gordon Manu Amponsah:** Investigation; resources; writing – original draft. **Henry Kofi Andoh:** Validation. **Kwadwo Faka Gyan:** Validation. **Yaw Amo Wiafe:** Conceptualization; investigation; writing – original draft; writing – review and editing.

## FUNDING INFORMATION

No funding was provided for this article.

## CONFLICT OF INTEREST STATEMENT

The authors have nothing to disclose with regard to commercial support.

## CONSENT

Written informed consent was obtained from the patient to publish this report in accordance with the journal's patient consent policy.

## Data Availability

Data sharing is not applicable.
